# Supplementary material for: Identification of photoperiod-induced specific miRNAs in the adrenal glands of Sunite sheep (Ovis aries)
Source: Front Vet Sci. 2022 Jul 22;9:888207. doi: 10.3389/fvets.2022.888207 (PMC9354845; doi:10.3389/fvets.2022.888207)
Supplement: Supplementary file 1 [file Data_Sheet_1.ZIP › Supplementary file/miRDeep2 file.zip!/miRDeep2 file/Novel_Structure/SPLP42A1/Novel_417.pdf]

Provisional ID	:	NC_019484.2_31887
Score total	:	2.3
Score for star read(s)	:	-1.3
Score for read counts	:	0
Score for mfe	:	2
Score for randfold	:	1.6
Score for cons. seed	:	
Total read count	:	27
Mature read count	:	27
Loop read count	:	0
Star read count	:	0

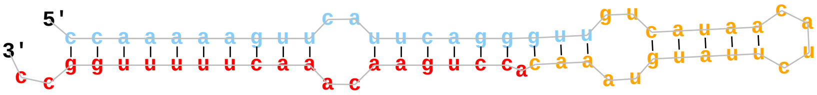

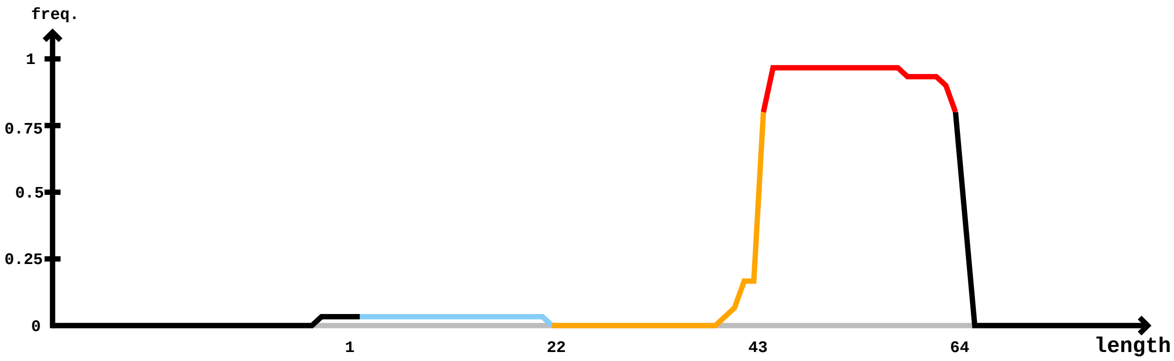

Star Mature

```
5'- ggaaaccucagaagacaaguaugggguuggccaaaaaguucauucaggguugucauaacaucuuauguaaacaccugaacaaacuuuuuggccaacccaauagauaaacaga   -3'    exp
    ...................(((.(((((((((((((((((..(((((((((..(((((....)))))..))).))))))..))))))))))))))))).))).........    reads      mm         sample
    ..........................uuggccaaaaaguucGuucagggu..............................................................    1          1          seq
    ....................................................................aaaAaccugaacaaacuuu.........................    1          1          seq
    .....................................................................aaAaccugaacaaacuuuuugg.....................    1          1          seq
    ......................................................................acaccugaacaaacuuuuuggc....................    3          0          seq
    ........................................................................aUcugaacaaacuuuuuggcc...................    18         1          seq
    ........................................................................accugaacaaacuuuuuggcA...................    1          1          seq
    .........................................................................ccugaacaaacuuuuuggcU...................    1          1          seq
    .........................................................................Ucugaacaaacuuuuuggcca..................    4          1          seq
```
